# Severe sepsis with Stevens Johnson syndrome caused by *Mycoplasma pneumoniae* – case presentation

**DOI:** 10.1186/1471-2334-14-S7-P62

**Published:** 2014-10-15

**Authors:** Monica Luminos, Mădălina Merişescu, Anca Drăgănescu, Angelica Vişan, Anuța Bilaşco, Cristina Negulescu, Endis Osman, Diana Slavu, George Jugulete

**Affiliations:** 1 National Institute for Infectious Diseases "Prof. Dr. Matei Balş", Bucharest, Romania

## Background

*Mycoplasma pneumoniae* is widely known as the etiological agent of “atypical pneumonia”, the most common clinical aspects of the infection being bronchiolitis and acute tracheobronchitis. It can also determine extra-pulmonary manifestation such as ear, nose and throat infections, neurological, cardiac or dermatological manifestations. Dermatological involvement is second most common, after respiratory infections, and it can vary from urticaria-like rashes to Stevens-Johnson syndrome. Stevens-Johnson syndrome is severe form of immune-complex–mediated hypersensitivity complex characterized by a hallmark of skin lesions spanning from mild forms to extensive involvement of skin and mucosa. It can be caused by a viral or bacterial infection or it can be drug induced.

## Case report

We present the case of an 8 year old male patient admitted in the Intensive Care Unit of the National Institute for Infectious Diseases "Prof. Dr. Matei Balş" with the suspicion of Stevens-Johnson syndrome. The patient’s personal history revealed that he had numerous episodes of upper or lower respiratory infections, all treated with antibiotics, and 2 episodes of ulcerative stomatitis and bullous pemphigus. The onset of the current episode was 6 days prior to admittance in our clinic with symptoms consistent with an upper respiratory infection for which his family doctor prescribed an antibiotic, under which his general state did not improve. After 4 days, the patient presented a generalized bullous eruption with consequent mucosal involvement.

Positive diagnosis was established through classic clinical and laboratory criteria and confirmed by serological methods which identified *Mycoplasma pneumoniae*. We have monitored the clinical and biological evolution under treatment. Evolution was slow but favorable, with limitation of cutaneous and mucous lesions and remission of respiratory symptoms.

## Conclusion

In this particular case, we considered two different factors concerning etiology: *Mycoplasma pneumoniae* and the administration of beta-lactam antibiotics, which could also have been the causative agent. Acute onset with respiratory symptoms was suggestive of an infectious etiology but, taking into consideration the patient’s previous allergic reactions and the onset after administration of beta-lactam antibiotics, we recommended precaution in case of future antibiotic treatments.

## Consent

Written informed consent was obtained from the parents for publication of this Case report and any accompanying images. A copy of the written consent is available for review by the Editor of this journal.

